# The comparison of family function based on the McMaster model in fertile and infertile women

**Published:** 2015

**Authors:** B Zanganeh, M Kaboudi, H Ashtarian, B Kaboudi

**Affiliations:** * School of Nursing and Midwifery, Kermanshah University of Medical Sciences, Kermanshah, Iran; ** School of Public Health, Kermanshah University of Medical Sciences, Kermanshah, Iran; *** School of Medical Science, Kermanshah University of Medical Sciences, Kermanshah, Iran

**Keywords:** family functioning, infertile women, infertility, McMaster model

## Abstract

**Introduction:** One of the main parts of the lives of infertile impaired is social relationships and their family functions. This study aimed to compare the family function based on the McMaster model in fertile and infertile women.

**Materials and Methods:** This research is a similar one. The population consisted of all infertile women referred to the two infertility centers and fertile females related to health areas located in the same area in Tehran. The sampling method was the convenience one and in both groups, 50 women were recruited as samples and they responded to the demographic and Family Assessment Device (FAD) questionnaires. The obtained information was investigated by using inferential and descriptive statistics and SPSS 22 software.

**Findings:** The results showed the following behavioral control variables (p = 0/ 003), roles (p = 0/ 002), emotional responsiveness (p = 0/ 020) and emotional involvement (p = 0/ 006). There was a clear distinction between fertile and infertile women and infertile women obtained worse scores.

**Conclusion:** The results indicated that infertile women have crucial problems in family functioning that can have an impact on other aspects of life and their health.

## Introduction

Medical Science and related areas have been known as the most advanced sciences especially in the recent decade, having developed dramatically. Although many of the diseases are treatable now, infertility is one of the diseases that has no individual treatment. Infertility was described as a beat to achieve pregnancy after a sexual action without protection after 12 months or more [**1**]. Lifetime prevalence of infertility in the world has been estimated between 6/ 6% and 26/ 4% and the average outbreak of a year of about 9 percent, only in 2007, 72/ 4 million persons in world being infertile. Also, in 2002, it was reported that impaired fertility has affected nearly eighty million persons from different sections of the world. Though the infertility rate is different in the various parts of world (5-30%), it was evaluated that nearly one-paired men/ women often have initial infertility or secondary one [**2**]. Several preliminary studies have shown that approximately one-third of the infertility cases are caused by diseases related to women, one-third because of men infertility, and one-third by a mix of issues in men and women simultaneously [**2**]. Researches were performed to investigate the etiology and treatment of infertility; some aspects of this disease having been neglected. In recent years, a change has been made in medical science and diseases are not monitored merely biomedical but are examined in a bio-psycho-social framework [**3**]. 

Presently, it is known that a variety of social and psychological problems appear in infertile couples. For example, high anxiety [**4**], low marital and sexual satisfaction [**5**], low marital adjustment [**6**], sexual dysfunction [**7**], eating disorders [**8**], lower sexual desire and arousal [**9**], more psychiatric disorders [**10**,**11**], reduced quality of life [**12**], and high depression prevalence [**4**,**13**,**14**] in infertile patients, have been reported. However, one of the most troubled parts of infertile patients’ lives is social relationships and family functions [**5**,**6**], and one of the most famous models in studying family functioning, is McMaster model of family [**15**,**16**]. 

Before the suggestion of the McMaster model, many models and theories were raised in the family area. For example, Bowen model emphasizes the concept of self-breakdown and the emotional system of the family structure. In the structural theory, family interactions are assessed and in the behavioral approach, they are emphasized on maladaptive behavior change of family members [**15**,**16**]. McMaster model of family functioning offered in the early 1960s at McMaster University by Epstein, Bishop, and Levin by combining the strengths of all previous models, provided a comprehensive approach in the field of family therapy. McMaster model evaluates the marriages and families. This model is based on a system theory and describes structure, organization, and exchange pattern of the marital unit, allowing family or marital relationships to be tested on a surface spectrum, from health to severe mental disorder. Although the McMaster model does not consider family functioning from the point of view of all the aspects, it is considered that the most important aspects that have the clinical appearance [**17**]. To understand the structure, organization and interaction pattern related to family, the model evaluated six dimensions of family life. The first dimension was the problem solving that represented the ability to solve problems at a level that will maintain an efficient operation of the family. The second dimension was the relationship that referred to the exchange of information between family members. The third dimension was the role that showed the efficiency of family methods in the distribution and performance of tasks. The fourth dimension or emotional responses referred to family members’ capability to appropriate emotions in comparison to different stimuli. The fifth dimension or the emotional involvement was referred to the amount of interest, attention, and investment of families about one another. Finally, the sixth dimension or behavioral control described the personal standards freedom [**15**,**16**]. 

Studies that have been performed by using this model have shown that family functioning is unsatisfactory in some families. For example, in families with different cultures [**18**], polygamous families [**19**], and families with a member with a mental illness [**20**-**25**] as well as in families with a member with physical problems and diseases [**26**], the family function is impaired. On the other hand, infertile women who have physical problems and disease [**1**] are consequently more likely to develop psychiatric problems [**10**,**11**], being at the same time at risk of causing familial dysfunction. According to a research, infertile women have unstable relationships [**27**] and more communication problems with their family [**28**]. The effects of infertility on relationships can also interfere with the operation of their families. On the other hand, the McMaster model is a comprehensive model of assessing the family function. This model has come together in different aspects of the married life, which refer to different approaches and has offered a comprehensive model in this area that can be useful in investigating family life in different groups, including in infertile women. 

To sum up, infertile women have many problems that may affect family relationships and may lead to many negative consequences including impairment of infertility treatment process and reduction of their mental health. On the other hand, although research on family and social relationships of these families has been done, these studies have not been based on a comprehensive model, including the McMaster model. Therefore, the present study was designed to compare family functioning in fertile and infertile women based on the McMaster model. 

## Materials and methods

**Sample and steps**

This study was a cross-sectional one. The population consisted of all infertile women cited to the two infertility centers and fertile women related to health areas located in the same area in Tehran. Among them, 50 infertile women and 50 fertile women were recruited by using the convenience sampling method and they completed the study questionnaires. For the ethical consideration, collected data and information from questionnaires were coded and the identified data were removed. Those with a severe impairment in the family functioning were advised to refer to mental health professionals.

**Research Tools**

A) Demographic questionnaire: The first tool that was used in this study was a questionnaire in which the demographic characteristics of the sample were recorded. In this questionnaire information such as age, the age of spouse, years of infertility, some referrals to treatment, education, and similar subjects were settled.

B) Family Assessment Device (FAD): FAD based on the McMaster theory and by Epstein, Baldwin, and Bishop has been developed. This questionnaire had 60 items and was scored from 1 to 4 based on the Likert scale. The validity of the questionnaires was appropriately evaluated within and outside Iran. In the Iranian version of the questionnaire, Cronbach’s alpha factor obtained was of 0/ 94 for all the instruments and the subscales were also defined as issue solving (0/ 86), communications (0/ 87), roles (0/ 87), emotional responsiveness (0/ 81), emotional inclusion (0/ 89), the general family functioning (0/ 82) [**29**]. In Yousefi’s study, the validity and reliability of the test were also examined. Cronbach’s alpha and Split-half for 60 items of the questionnaire were obtained; 0/ 83 and 0/ 82, respectively. The convergent and divergent validity of the questionnaire with the communication patterns questionnaire harnessed the center questionnaire for the subscales of emotional response and merged with the other, obtaining the values 0/ 46, 0/ 36, -0/ 41, -0/ 43, respectively [**30**]. In the study of Mohammadi and Molkkhosravi, Cronbach’s alpha for the entire scale was 0/ 90 and the test-retest coefficient, was 0/ 82 [**31**].

**Statistical analysis methods**

SPSS 21 software was used for data analysis. In the inferential statistics, independent t-test and Mann-Whitney U-test were also employed to evaluate the dimensions of family functioning in infertile and fertile women. 

## Results

In the current research, 50 fertile women and 50 infertile women participated. The mean age of fertile and infertile women, as well as the women’s husbands ages and duration of the marriage, are presented in **Table 1**.

**Table 1:** Average and nominal age deviation, age of husband and duration of marriage in 2 teams of infertile and fertile women

Infertile		fertile		variable
standard deviation	Mean	standard deviation	Mean	Age husband age duration of marriage
53/5	90/30	85/5	70/28	
31/5	48/33	05/6	08/32	
10/4	84/6	88/2	78/5	

As shown in **Table 1**, the average age of fertile women was 28/ 70 and average age of infertile women was 30/ 90. Also, the fertile women’s husband age was 32/ 8 and the infertile women’s husband age was 33/ 48. The average marriage duration in fertile women was 5/ 78 and in infertile women, it was 4/ 84.

Other demographic variables of the sample group including education, husband’s education, occupation, husband’s job, disease history, husband’s disease history, smoking, husband’s smoking history, and income are shown in **Table 2**. 

**Table 2:** Repetitive and ratio of demographic variables in infertile and fertile women

Infertile		fertile		group	variable
percentage	Frequency	percentage	Frequency		
14	7	8	4	illiterate	Education
26	14	26	13	High school	
44	15	44	22	diploma	
22	14	22	11	Bachelor	
6	3	12	6	illiterate	Husband’s Education
20	10	12	6	High school	
46	23	34	17	diploma	
28	14	42	21	Bachelor	
78	39	76	38	unemployed	job
10	5	12	6	self-employed	
12	6	12	6	government job	
72	36	62	31	Unemployed and self-employed	husband's job
28	14	38	19	government job	
16	8	26	13	yes	history of disease
84	42	74	37	no	
18	9	12	6	yes	Husband’s history of disease
82	41	88	44	no	
14	7	10	5	yes	smoking
86	43	90	45	no	
46	23	50	25	yes	Husband’s smoking history
54	27	50	25	no	
18	9	24	12	good	income
58	29	50	25	moderate	

**Fig. 1:**
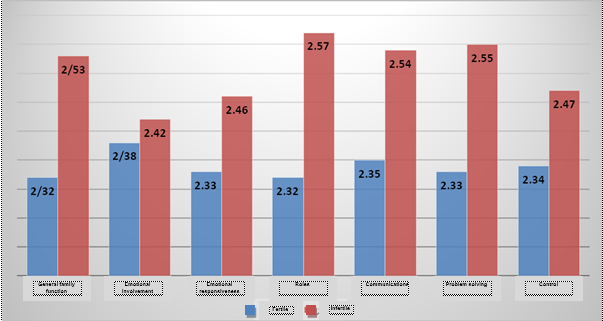
Mean score of fertile and infertile women in the Family Assessment Device (FAD)

**Fig. 1** shows the mean scores of fertile and infertile women in the Family Assessment Device (FAD). As shown in this figure, in all the scales and the sum rate of the questionnaire, infertile women have higher scores, which mean a worse family functioning (**Fig. 1**).

Because the normal distribution of data of the control subscale of Family Assessment Device (FAD), the comparison between fertile women and infertile women analysis of the t-test was used for other subscales that were not distributed normally. Mann-Whitney U test was also used and its findings are presented in **Table 3**.

**Table 3:** Variable t test and Mann-Whitney U test which compares the control in fertile and infertile women

P	Z	infertile		fertile		variable
		standard deviation	mean	standard deviation	mean	
003/0	08/3	26/0	53/2	41 /0	32/2	Behavior control*
564/0	577/0	41/0	42/2	46/0	38/2	Problem solving**
066 /0	835/1	28/0	46/2	38/0	33/2	
002 /0	055 /0	37/0	57/2	42 /0	32/2	Roles**
020 /0	323/2	43/0	54/2	40/0	35/2	Emotional responsiveness**
006/0	734/2	35/0	55/2	41/0	33/2	Emotional involvement**
100/0	64/1	33/0	47 /2	43 /0	34/2	General performance**

As shown in **Table 3**, the mean score of the control of infertile women obtained 20/ 88 with a standard deviation of 3/ 71 and in infertile woman 22/ 80 with a standard deviation of 2/ 36, this distinction between the 2 teams being statistically clear (p = 0/ 003). Given that the individuals who obtained higher scores in the Family Assessment Device (FAD) were worse in family functioning, infertile women had a worse situation than fertile women in the family functioning control variable. Also, in variables of problem solving (p = 0/ 564], roles (p = 0/ 066) and general function (p = 0/ 100), no clear variation was found between the 2 teams of fertile and infertile women. In the variables of roles (p = 0/ 002), emotional responsiveness (p = 0/ 020) and emotional involvement (p = 0/ 006) showed a clear distinction between the 2 teams of infertile and fertile females. These findings showed that in these variables, infertile women had higher grades and therefore more problems in family functioning. 

## Discussion and conclusion

As it was observed in the results part in four subscales of the family functioning including behavioral control, roles, emotional responsiveness and emotional involvement between fertile and infertile women, a significant difference was observed, and infertile women obtained worse scores. In the three subscales of problem solving, communication and general family functioning, there was not a clear distinction between the 2 teams.

There is no study about the family functioning of infertile women performance, and this may be the first study of this kind. However, two types of studies can be considered related to the study. The first studies are those in which the family aspects of infertile women have been examined and variables such as family functioning have been investigated.

Jo May, and his colleagues (2012) investigated social relationships of infertile women and found that infertile women had poor relations with the members of their family and more likely considered their marital intimacy inappropriate [**28**]. In the Monga and colleagues study in 2004, it was shown that the marital adjustment in infertile women was significantly lower than in fertile women [**6**]. In another study conducted in 1992 by Benzon and his colleagues in Canada, results revealed a significant decrease in the marital adjustment of infertile couples [**32**]. 

In addition to the preceding studies, some research that indicated a dysfunction in the families in which a family member had a serious problem was also available. These results may be extended to infertile families. For example, Mirzaee and colleagues found in their research that there was a significant difference between groups with an addicted member with other families, in all the aspects of family functioning [**20**]. Another study undergone in 2010 also was conducted in Iran by Bayrami and colleagues and showed that depressed patients regarding the general functioning, communication, emotional responsiveness and emotional involvement had lower scores than healthy individuals but regarding the problem-solving, behavioral control and role no significant difference was found [**15**]. Another study was performed in 2010, in Iran by the Qolizadeh and colleagues, which compared the family function of obsessive-compulsive patients with the healthy subjects. At the end of the study, it was found that OCD patients are weaker than normal people in many aspects of family functioning. OCD patients showed a lower efficiency in the general functioning, communication, and emotional engagement compared to healthy subjects [**16**].

However, the question here is what causes family dysfunction in infertile individuals? The family functioning is the concept of exchange pattern in the marital unit that involves issue solving, association, mental responsiveness, feeling involved, and behavioral management. For instance, in the case of the variability of roles, there is an expectation in the couples that after a while, the role of the wife or husband has to be transferred to the father–mother role. This transfer does not occur in infertile couples and ultimately, their expectations about their roles are questioned. For example, some theorists believe that the women leave a place empty for maternity when introducing their identity, and if this role is not in their lives, many psychological problems may appear [**33**].

Some explanations are also provided by experts in the case of emotional responsiveness and emotional involvement. For example, Sherrod believes that infertility has many emotional aspects, and hence, attention to the emotional aspect of infertility is critical [**34**]. Given that one of the reasons of every man and woman in marriage is expected to give birth to a child during the marital life, failure to achieve this aim causes the loss of relationships between them [**35**].

So, the problems of infertile couples can be caused by emotional ties they feel about themselves and their relationship.

Another variable that was observed in infertile women as compared to fertile women was the behavior control. Behavior control can be considered a method of family criteria that is used to control the behavior of its members, such as the physical risk, biological and motivational needs like eating, sleeping, instinct, sex, and aggression, social behavior that includes the behavior inside and outside of the family.

There are four methods to control the behavior: strict control, flexible control, control of non-interference and chaos control [**36**-**39**]. According to the findings of this research, behavioral control is poor in infertile families. Those are some of the needs of people in this kind of marital relationship. Due to the expectations of change in their relationships and on the other hand, due to not meeting these expectations, they encounter some problems in balancing the behavior control. Examples of these problems have been observed in previous preceding studies [**6**,**32**].

Generally, it can be said that today all experts believe that physical illnesses have psychological and social aspects. Hence, infertility is considered a bio-psycho-social crisis as well [**40**]. Thus, the probability of the development of psychological and family disorders in infertile patients is more prevalent than in the other groups, and necessary steps should be taken in this regard.

## Conclusion

This study was the initial research of the investigation of family functioning in infertile families and was evaluated via the family function of fertile individuals. In this study, the results showed that in all subscales of the family functioning, infertile women obtained worse scores, although only in four subscales there was a clear variation among fertile and infertile women. The results also indicated that infertile women have poorer family functioning in comparison with normal individuals and specific groups such as people with OCD. The results indicated that in general, infertile families have many problems in family functioning such as the division of roles, emotional communication, and behavior control. The results of this research can be used by authorities for future planning. For example, counselors and psychologists can be deployed in infertility centers to screening people with family problems from the beginning and perform early interventions to improve family problems. Such programs may also have long-term effects such as the mental health of infertile couples and may reduce divorce.

**Limitations and research recommendations**

Any research has some limitations and this study makes no exception. The reasons for this limitation can be related to the lack of a complete control of human, physical limitations, and hardware limitations or restrictions of time and space that most of them refer to.

The negative attitude of infertile women and particularly of their spouses towards the study is highlighted in the filling of the questionnaires, lack of full control of confounding variables such as personality, physical and mental variables, as well as socio-economic and cultural variables. Lack of proper research facilities, including a study room, suitable chairs, and other facilities in infertility treatment centers, limited the ability to generalize results due to a limited study sample of infertile women as well as a province of the country.

Due to the researcher, his study has fully engaged in the subject matter and tried to discover the strengths and weaknesses of his research and others so that he could offer proper and applied proposals. Some suggestions are mentioned here. It was recommended due to abundant problems in the field of family functioning of infertile individuals, other aspects of their family life being examined as well.

It is recommended that this research is repeated at broader dimensions regarding gender, geography and culture. It is also recommended that interventions are designed for infertile couples so as to target the family function of this group and their effectiveness evaluation by the researchers. Given the high level of family functioning problems of infertile families variables of family functioning, it is suggested that interventions are performed to improve the patient’s family circumstances. It is suggested in the infertility treatment centers, counselors or psychologists are deployed so that families suffering from family function problems are identified and treated faster.
